# Indigenous inoculant dampens the impact of remediation of heavy metal polluted soil on arbuscular mycorrhizal fungal communities

**DOI:** 10.1007/s00572-026-01268-1

**Published:** 2026-05-09

**Authors:** Nataša Šibanc, Dave R. Clark, Marjetka Suhadolc, Domen Leštan, Alex J. Dumbrell, Irena Maček

**Affiliations:** 1 Department of Forest Physiology and Genetics, Slovenian Forestry Institute, Ljubljana, Slovenia; 2 School of Life Sciences, University of Essex, Colchester, UK; 3 Institute for Analytics and Data Science, University of Essex, Colchester, UK; 4 Department of Agronomy, Biotechnical Faculty, University of Ljubljana, Ljubljana, Slovenia; 5 Department of Biology, Biotechnical Faculty, University of Ljubljana, Ljubljana, Slovenia

**Keywords:** AMF, Arbuscular mycorrhiza, Agroecosystems, Biodiversity, Urban soil, Fungal traits

## Abstract

The finite resource of fertile soil can take thousands of years to develop. Non-degradable toxic metals (heavy metals), pose a significant health risk due to their persistence in the environment. Soil washing with EDTA (ethylenediaminetetraacetic acid) has been shown to remove toxic metals (Pb, Zn, Cd), but this process can also have a significant impact on soil microbial diversity. In this study, we investigated, using molecular methods (18S rRNA amplicon sequencing), the development of the arbuscular mycorrhizal (AM) fungal communities in the roots of *Lolium perenne* after a soil remediation treatment with EDTA. Plants were grown on two soil types (calcareous and acidic), in remediated and control (unremediated, polluted) soils, and with or without the addition of a local environmental inoculant (grassland soil and roots). The addition of the inoculant increased the richness and evenness of AM fungi, and reduced the compositional dissimilarity of communities between remediated and unremediated soils. Disturbance from the remediation process and inoculation, lead to a shift in the identity of dominant taxa reflecting different traits among the AM fungi. This suggested two main mechanisms behind community development in remediated soils, i.e. priority effects and competitive exclusion among dominant taxa. These findings emphasise the importance of nature-based solutions (indigenous environmental inoculants) and AM fungi for sustainable soil management practices. This is particularly important for restoring soil biodiversity in sites that have been exposed to multiple disturbances, which may include contaminated sites and soil remediation, as well as conventional agricultural systems, and urban soils.

**Supplementary Information:**

The online version contains supplementary material available at 10.1007/s00572-026-01268-1.

## Introduction

Fertile soil, a finite and valuable resource, is a slow-forming medium that takes millennia to develop. Soils contaminated with non-degradable, potentially toxic metals, known also as heavy metals, are a significant concern due to the persistence of heavy metals in the environment (Amundson et al. 2015). These contaminated soils are often rendered unsuitable for their intended purposes and pose a serious threat to human and environmental health (Hartley and Lepp 2008). Numerous countries, including Slovenia, have areas of arable land contaminated with heavy metals from industrial activities in the 20th century (EEA, 2020; FAO, 2021). In Slovenia, the Meza Valley afflicted by lead (Pb), zinc (Zn), and cadmium (Cd) contamination spans roughly 50 km² in the northern region of the country, and incorporates a decommissioned lead mining and smelting industrial facility. The unique topography, combined with five centuries of emissions from the mineral extraction industry, has led to the widespread contamination of soils throughout the area (Finzgar et al. 2014). Urban and rural areas, despite heavy metal contamination, still engage in activities such as gardening and food cultivation on polluted soils, putting vulnerable populations, particularly children, at risk of serious health issues (Jez and Lestan 2015). This study focused on two types of soil (calcareous and acidic soil) contaminated with lead, cadmium and zinc, to assess the impact of EDTA-based remediation techniques on soil microbial communities. Specifically, we focus on plant symbiotic arbuscular mycorrhizal (AM) fungi, and follow the development of their communities in the remediated soil.

Soil biodiversity plays a crucial role in maintaining soil health and regulating soil functions (Delgado-Baquerizo et al. 2016; Griffiths and Philippot 2013). Soil fungi, in particular, are vulnerable to mechanical and chemical disturbances due to their filamentous nature, with their communities being frequently affected by activities like soil tillage (Helgason et al. 1998; Oehl et al. 2010). The ancient and ubiquitous group of AM fungi (Glomeromycotina/Glomeromycota) (Spatafora et al. 2016; Tedersoo et al. 2018), that forms symbiotic relationships with over 70% of plant species (Brundrett and Tedersoo 2018), including crops and fruit trees, are of particular interest in arable soil due to their potential role in sustainable food production and agriculture. The benefits provided by this symbiosis, such as improved nutrient and water uptake and protection from pathogens, depend on the specific interactions between the AM fungi and their host plants, as the identity and diversity of AM fungal symbionts strongly affect the functioning of mycorrhiza (Blažková et al. 2021).

Soil washing with EDTA (ethylenediamine tetraacetic acid), a chelating agent, has been shown to effectively remove toxic metals (Pb, Zn, Cd) from polluted soils (e.g. Finzgar and Lestan, 2007; Pociecha and Lestan 2012; Voglar and Lestan 2013), but this process can also have significant effects on soil microbial activity (Jelusic and Lestan 2014; Suhadolc et al. 2026), particularly on fungal communities (Maček et al. 2016, 2022; Kaurin et al. 2021). Studies have demonstrated the negative impact of EDTA soil washing on symbiotic AM fungi, with long-lasting effects on their colonisation of plant roots (Kaurin et al. 2021; Maček et al. 2022). However, within outdoor gardens, there is evidence of the potential for recovery of AM fungal communities in remediated soils (Kaurin et al. 2021; Maček et al. 2022). In a study conducted in a garden setting with vegetable beds, researchers examined the effects of soil treated with EDTA on various enzyme and microbial indicators, including the colonisation of roots by AM fungi (Kaurin et al. 2021). While traditional gardening practices were successful in restoring the biological functions of the treated soil, the most significant and lasting negative impact was observed on the symbiotic AM fungi. Based on the abundance of the total fungal community (qPCR targeting ITS genes) and root colonisation with AM fungi we have also demonstrated the recovery potential of fungal communities in remediated soils, showing the importance of plant presence in this process (Maček et al. 2022). Although there was some recovery in fungal colonisation of roots in the above studies, there is currently a lack of data on the composition and diversity of AM fungal communities in treated systems. There are also no data on the impact of specific remediation techniques tailored to different soil types or the use of different inoculants to accelerate mycorrhiza formation. The latter is particularly important in the light of recent findings that many AM fungal commercial inoculants are inefficient, can lack quality control, contain pathogens and have negative impacts on native AM fungal communities and crop growth (e.g. Hart et al. 2018; Vahter et al. 2023; Koziol et al. 2024).

Here we take a crucial step towards understanding the recovery of AM fungal communities in remediated soils, actions that can improve it (e.g. inoculation with indigenous inoculants) and emphasise the importance of AM fungi in sustainable soil management practices and regenerating soil diversity using nature-based solutions. In the study, we used soil mesocosms to examine the diversity and development of the AM fungal community in roots of *Lolium perenne* L., grown on two soil types (calcareous and acidic) in polluted (unremediated) and remediated soils after an EDTA washing treatment, and with or without the addition of local environmental (indigenous) inoculant (grassland rhizosphere soil and roots). We test the following hypotheses: (H1) EDTA washing (remediation) and inoculation affect the composition of AM fungal communities in different soil types in different ways, resulting in distinct AM fungal communities with a lower level of community compositional effects after remediation when adding the inoculant. (H2) Remediation reduces (AM fungal) taxon richness, whereas soil inoculation increases taxon richness and evenness across all remediated and contaminated soils, with diversity increasing through time since remediation. (H3) Disturbance (remediation procedure) and inoculation lead to a shift in dominant taxon identity, reflecting different traits among the AM fungi in investigated soils.

## Materials and methods

### Experiment set-up and soil remediation

Soils used for the mesocosm experiment were collected from the top 30 cm layer from a vegetable garden in Mežica, Slovenia (SI – calcareous soil) and a farmland in Arnoldstein, Austria (AT – acidic soil). The initial content of Pb, Cd and Zn before remediation (Table S1) was higher in SI soil with 1131.2 ± 27.12 mg kg^− 1^ Pb, 6.74 ± 0.08 mg kg^− 1^ Cd and 755.97 ± 14.75 mg kg^− 1^ Zn (mean ± SE), while AT soil had 756.04 ± 12.09 mg kg^− 1^ Pb, 4.28 ± 0.07 mg kg^− 1^ Cd and 452.95 ± 7.06 mg kg^− 1^ Zn. As pH is the most influential parameter for metal availability in soil, pH-neutral - calcareous (SI) and acidic (AT) soils were selected (pH 6.9 vs. pH 5.0, respectively).

Half of the soils from both sites was remediated by washing using EDTA based technology (the ReSoil^®^) as described by Lestan (2017), and Gluhar et al. (2021); using 60 mM Ca-EDTA kg^− 1^ for the AT soil and 100 mM Ca-EDTA kg^− 1^ for SI soils (remediated soil), and the other half was left untreated (unremediated soil). The content of Pb, Cd and Zn after remediation (Table S1) was also higher in SI soil with 479.54 ± 13.86 mg kg^− 1^ Pb, 3.07 ± 0.15 mg kg^− 1^ Cd and 513.64 ± 20.84 mg kg^− 1^ Zn (mean ± SE), while AT soil had 202.72 ± 1.25 mg kg^− 1^ Pb, 0.83 ± 0.06 mg kg^− 1^ Cd and 262.08 ± 2.22 mg kg^− 1^ Zn. The untreated and remediated soils from both sites were placed in 35-litre mesocosms (32 mesocosms; 24 cm diameter, 42 cm height) on June 23rd (2016), filled with a 5-cm layer of quartz sand (1–3 mm) at the bottom, and covered with a plastic mesh (0.2 mm).

The contaminated calcareous and acidic soils were examined in the experiment according to two factors: the first factor was remediation with two levels: no remediation (unremediated), remediation (remediated). The second factor was inoculant (uncontaminated semi-natural grassland rhizosphere soil with plant roots): absence (without inoculant) or presence of inoculant in the soil (with inoculant). Each treatment was replicated in four mesocosms (*n* = 4).

Mesocosms were planted with 70 ml of sterilised (2 min in 10% bleach) plant seeds (*Lolium perenne* L.) on July 11th (2016). We added environmental inoculant (rhizosphere soil and roots) to the mesocosms as treatments (16 mesocosms) to determine its effect on soil microbial communities (100 ml inoculant in each mesocosm of the treatment). The rhizosphere inoculant was taken from uncontaminated semi-natural grassland in Ljubljana (Slovenia) and mixed only into the top 5 cm of the soil layer in the mesocosms.

### Soil and plant sampling

Soil samples were collected at the beginning of the mesocosm experiment, just before the soils were placed into the mesocosms (June 2016). Four months after plants seeds and inoculum were added to mesocosms (November 2nd, 2016), plants were collected for measurements of heavy metal content (Table S2). Soil samples were dried at 40 °C for 24 h and sieved through a 2 mm mesh sieve to remove roots and stones. Sieved soils were then used for soil chemo-physical analyses and heavy metals content measurements. Plant samples for biomass measurements were collected from mesocosms, homogenised, and oven dried at 40 °C for 24 h (Table S3).

### Plant root sampling

Plant roots were sampled for AM fungal colonisation on August 2nd and November 3rd (2016), June 6th and November 18th (2017), and April 5th and June 4th (2018). In addition, three root samples were collected from the inoculant. Roots were collected in a 3 × 15 cm soil core from each mesocosm, washed with tap water, and dried at 70 °C, before being stored at room temperature. Results on plant root AM fungal colonisation are available in Maček et al. (2022).

### Soil analyses

For soil analyses, samples were air-dried and sieved to 2 mm (ISO11464, 2006). Total metal contents (Pb, Cd and Zn) were measured using inductively coupled plasma mass spectrometry (ICP-ES/MS) after digestion in aqua regia (Bureau Veritas Mineral laboratories, Canada). Soil organic carbon (SOC) and total nitrogen (TN) were determined by dry combustion (ISO 10694, 1996; ISO 13878 1995) using elemental analyser (Elementar vario MAX instrument, Germany). Carbonates were determined manometrically after soil reaction with HCl (ISO 10693 1996) and soil texture by the pipette method (ISO 11277, 2009). Soil pH was measured in a 1/2.5 (w/v) ratio of soil and 0.01 M CaCl_2_ suspension (ISO 10390 2005).

### Molecular methods for AM fungal community assessment

To quantify the AM fungal community, mixed plant roots were homogenised using a Retsch mixer mill (Retsch). DNA was extracted from a 50 mg dry subsample of the homogenised roots using DNeasy Plant Pro DNA isolation kits (Qiagen), following the manufacturer’s instructions. AM fungal communities were quantified using Illumina MiSeq NGS of amplicons of the SSU (small subunit) rRNA gene, which is a frequently used marker gene in studies of AM fungal diversity (Cotton et al. 2014; Davison et al. 2015; Dumbrell et al. 2010a; Maček et al. 2011).

To produce amplicon libraries for Illumina MiSeq NGS, a 550-bp fragment of the SSU rRNA gene was first amplified by PCR using AppTaq RedMix (2X) (Appleton Scientific), the universal eukaryotic primer NS31 (Simon et al. 1992) and the primer AM1, which excludes plants, amplifies the major AM fungal families (Helgason et al. 1998) and provides accurate repeatability with no detectable PCR biases (Cotton et al. 2014). Forward and reverse primers were modified to contain Illumina-specific overhang adapter sequences (Sigma). PCR was carried out in a 25 µl reaction volume with 1 µl of DNA template, 12.5 µl of AppTaq RedMix (2X), 0.05 µl T4 Gene 32 Protein (Invitrogen) and 2 µM of each primer (PCR conditions: 95 °C for 3 min; 30 cycles at 95 °C for 15 s, 64 °C for 15 s and 72 °C for 15 s; and 72 °C for 10 min) on a Applied Biosystems Veriti Thermal Cycler (Thermo Fisher Scientific). PCR products were purified using Agencourt AMPure XP magnetic beads (Beckman Coulter). A secondary indexing PCR was then used to attach Illumina sequencing adapters and multiplex indexes, using the Nextera XT Index Kit (Illumina) and following Illumina’s recommended protocols. Secondary PCR products were purified using Agencourt AMPure XP magnetic beads (Beckman Coulter), before quantification using a PicoGreen Assay on a FLUOstar Omega Microplate Reader (BMG Labtech). Equimolar concentrations of 159 successfully amplified samples were pooled and sequenced on an Illumina MiSeq with V3 2 × 300 bp paired-end chemistry at University of Essex, UK.

### Bioinformatics

First, we stripped primers from the sequences using cutadapt 4.4 (Martin 2011), discarding any sequences with mismatches in the primer sequence. Forward and reverse sequences were then quality filtered, trimmed, and pair-end aligned using fastp v 0.21.0 (Chen 2023). Here, sequences where more than 20% of the read fell below Q20 were filtered and a ‘sliding-window’ approach was used to trim sequences, moving in the 5’ to 3’ direction and trimming where the average quality drops below Q20, retaining only those sequences longer than 200 nucleotides after trimming. Sequences passing the quality filtering and trimming steps were then pair-end aligned with a minimum detected overlap of 15 base pairs. We implemented an additional post-overlap length filter using Bash functions to retain sequences between 500 and 527 nucleotides in length. We then combined all sequences across all samples and dereplicated sequences with VSEARCH v 2.7.1 (Rognes et al. 2016). Remaining sequences were then denoised and clustered into OTUs at 97% similarity with VSEARCH. We used an OTU-based approach as there are multiple recognised issues with applying an amplicon sequence variant approach on SSU sequences (see Kauserud 2023). Centroid sequences were chimera checked, using the ‘uchime3’ algorithm implemented in VSEARCH, and sequences were then mapped to remaining centroids at 97% similarity to create an OTU table. We removed non-AM fungal OTUs by assigning taxonomy against the SILVA nr database (v132), keeping only those OTUs identified as Glomeromycetes. To assign taxonomy at a finer resolution, we queried OTU centroid sequences against the MaarjAM database (Opik et al., 2010) using BLAST (Altschul et al. 1990), assigning each OTU to a virtual taxon (VT) identifier.

### Data analyses

The OTU table was imported into R (version 4.40; R core team, 2024) and randomly rarefied to a depth of 1,152 sequences per sample, discarding any samples with fewer sequences (*n* = 14). Alpha-diversity for each sample was then calculated as OTU richness and evenness (inverse Simpson’s index) using functions from the Hill R package (Li 2018). Changes in community richness and evenness across the course of the study and between treatments were then quantified using negative binomial generalised linear models (nb-glms) or linear mixed-effects models using the MASS and lme4 packages (Venables and Ripley 2002; Bates et al. 2015). Pairwise beta-diversity was quantified using Sorensen’s index as implemented in the betapart R-package (Baselga and Orme 2012) and visualised using NMDS analysis. Location and dispersion effects on beta-diversity between experimental treatments were analysed using PERMANOVA (adonis2) and permutational homogeneity of variances test (betadisper) from the vegan R-package (Oksanen et al. 2025). Rank-abundance curves were calculated using data.table functions. All data visualisations were constructed using the ggplot2 and patchwork R-packages (Wickham 2016; Pedersen, 2024).

## Results

We analysed 3,061,284 sequences across 187 samples out of a total 3,276,857 raw sequences from an initial 201 samples once reads that did not meet our quality control criteria, and samples with < 1152 sequences were removed (see the Methods section for more details). The remaining sequences comprised a total of 55 OTUs (GenBank accession number BioProject ID PRJNA1347864), 36 of which were identified as Glomeromycetes. Rarefaction curves (Fig. S4) showed communities were sufficiently deeply sequenced to capture most AM fungal diversity (after communities were rarefied to an even depth 1,152 reads per sample). The OTUs were assigned to 33 unique MaarjAM virtual taxa (VT), with a minimum 94.6% identity (1st quartile = 98.1, median = 99%, 3rd quartile = 99.4%).

### AM fungal community composition

**Fig. 1:**
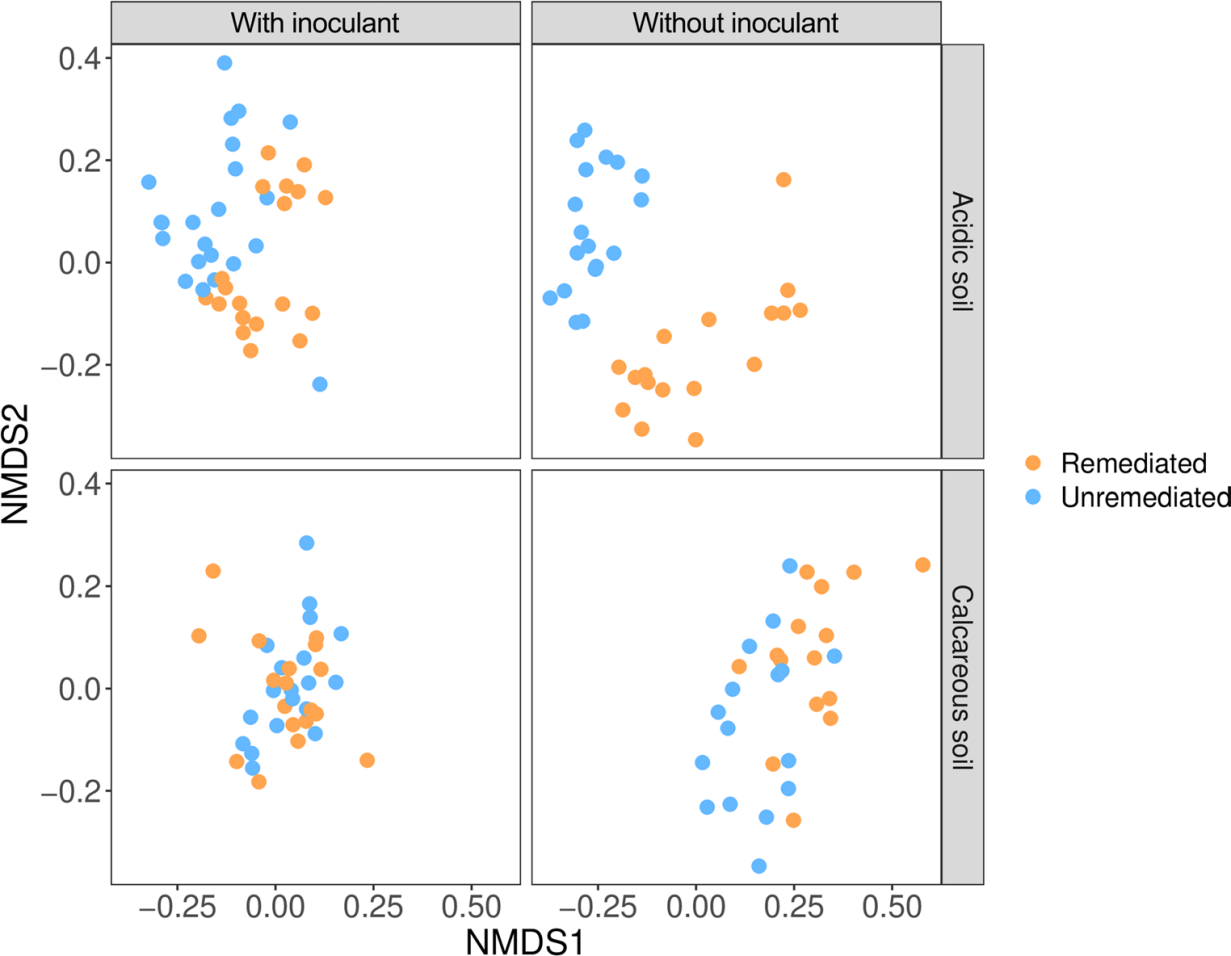
Non-metric multidimensional scaling (NMDS) plot of AM fungal community structure based on Sorensen dissimilarity. Each point represents a single community, with points closer together indicating more compositionally similar pairs of communities

Across polluted soils (acidic and calcareous), AM fungal community composition differed significantly between both remediation and inoculation treatments. In acidic soils, remediation had a large effect on community composition, accounting for 32% of the compositional variation (PERMANOVA; F_1, 73_ = 41.16, R^2^ = 0.32, *P* < 0.001), whilst the addition of inoculant explained a further 8% of the variation (F_1, 73_ = 10.64, R^2^ = 0.08, *P* < 0.001), and the interaction of remediation and inoculant addition contributing a smaller amount of explained variation (F_1, 73_ = 5.67, R^2^ = 0.04, *P* < 0.01). In contrast, the addition of inoculant had the greatest effect on composition in calcareous soils (PERMANOVA; F_1, 65_ = 21.68, R^2^ = 0.20, *P* < 0.001), with lesser effects from remediation (F_1, 65_ = 11.42, R^2^ = 0.10, *P* < 0.01) and the interaction of remediation and inoculant addition (F_1, 65_ = 12.90, R^2^ = 0.12, *P* < 0.001). Notably, the addition of inoculant not only reduced the compositional dissimilarity between remediated and unremediated calcareous soils but also reduced the dispersion of compositional variation in remediated soils (F_3, 65_ = 4.16, *P* < 0.01) (Fig. 1), suggesting that inoculation with a ‘native’ soil type can effectively minimise compositional effects of remediation. There was no difference in compositional dispersion between remediation or inoculation treatments for acidic soils (F _3, 73_ = 1.02, *P* = 0.39).

### AM fungal diversity

**Fig. 2:**
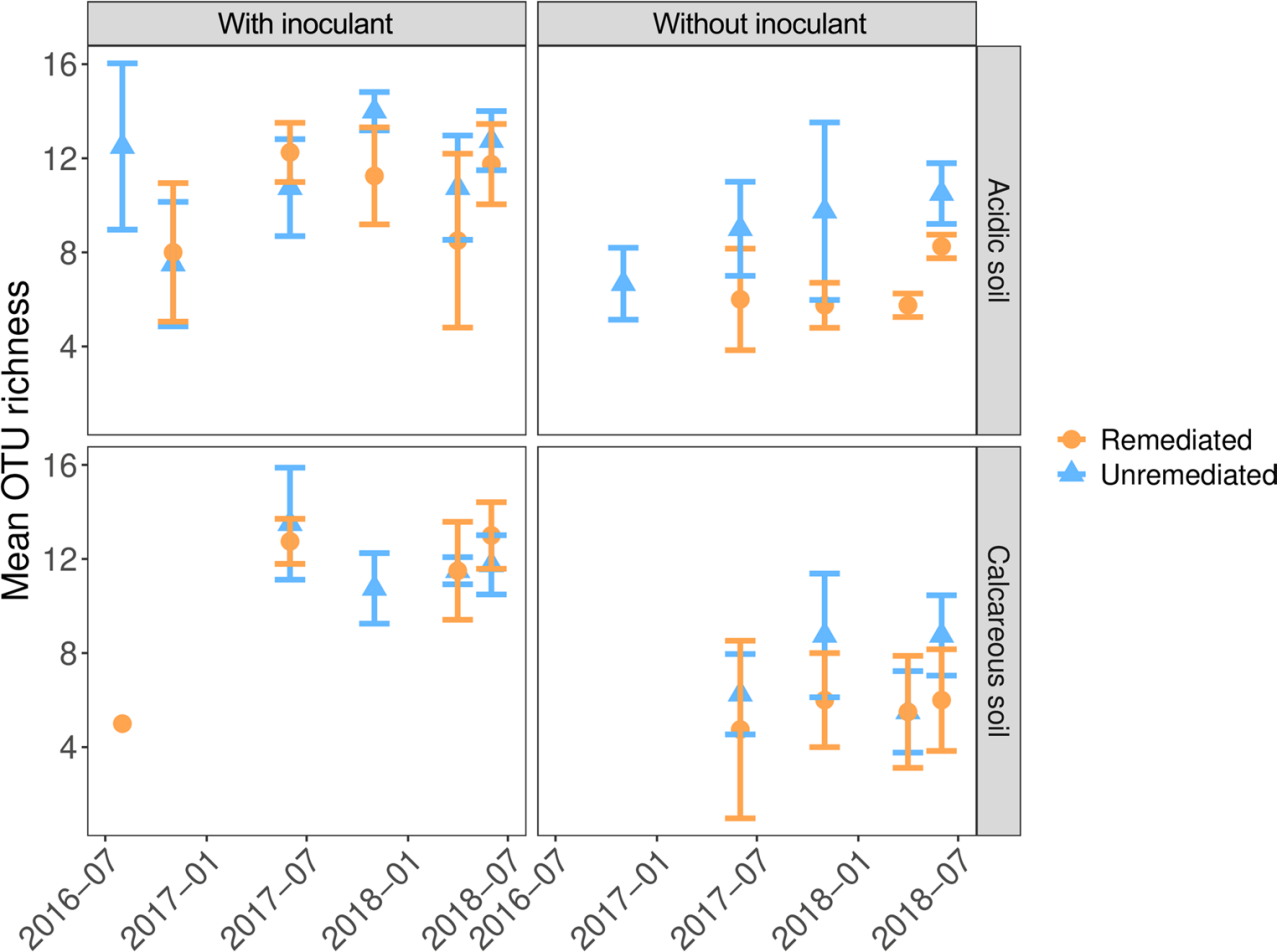
Temporal dynamics of operational taxonomic unit (OTU) richness of AM fungal communities in heavy metal contaminated soils subject to remediation and inoculation with local inoculant

The effect of soil remediation on AM fungal OTU richness (Fig. 2), was moderated by the inoculation treatment. In acidic soils without inoculant, remediation reduced OTU richness (coef = 0.66, z = -3.48, *P* < 0.001). In contrast, the addition of inoculant nullified richness differences between remediated and unremediated soils (coef = 0.89, z = -1.37, *P* = 0.17) and increased the richness across both soil treatments by ~ 43% (coef = 1.43, z = 4.66, *P* < 0.001). In the polluted calcareous soils, remediation had no effect on OTU richness in either inoculation treatment (without inoculant; z = -1.50, *P* = 0.13, with inoculant; z = -0.32, *P* = 0.75), although inoculation still reduced the difference in OTU richness of remediated and unremediated soils (without inoculant; coef = 0.81, with inoculant; coef = 0.97).

**Fig. 3:**
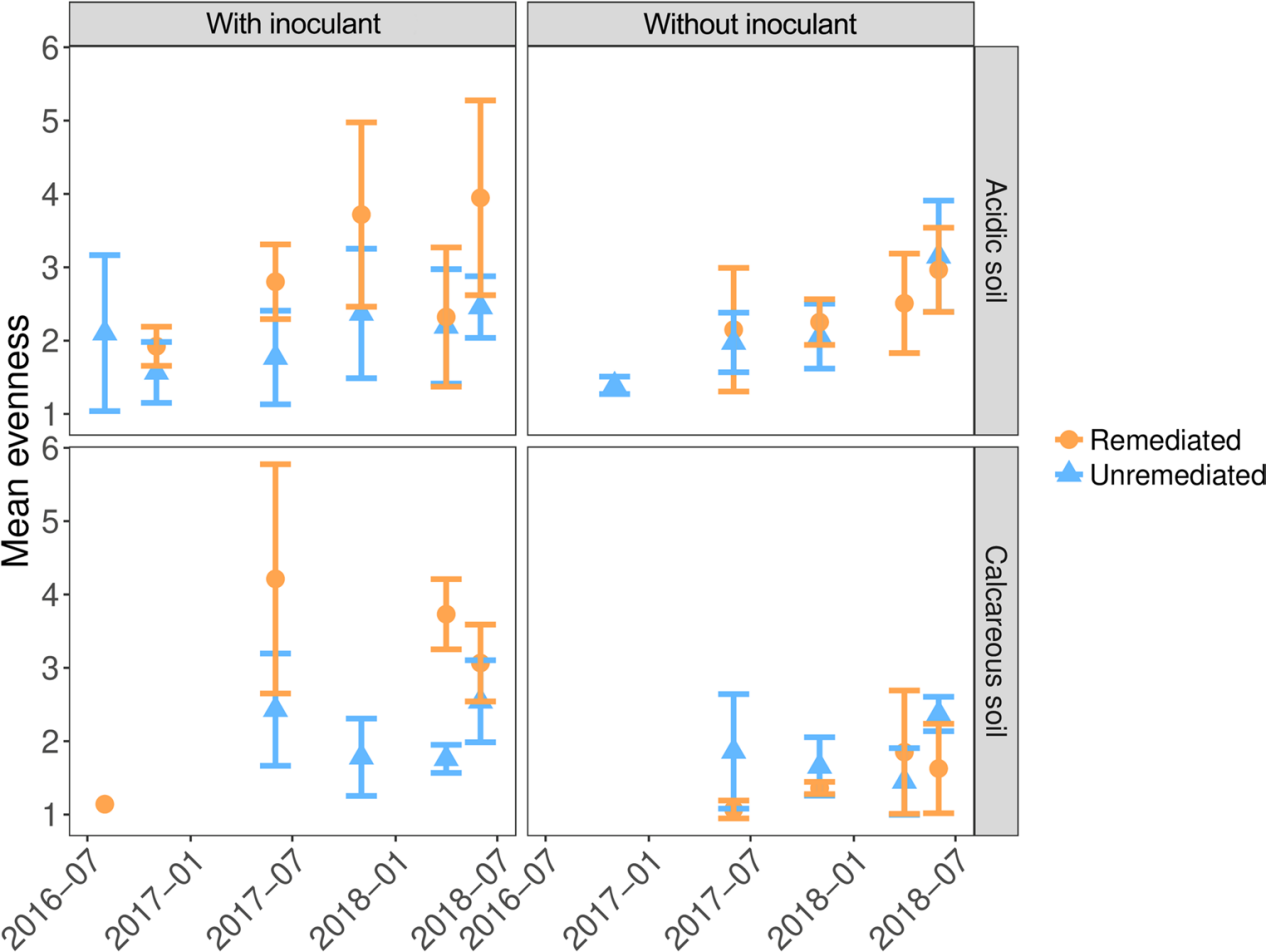
Temporal dynamics of the evenness of AM fungal communities in heavy metal contaminated soils subject to remediation and inoculation with unpolluted soil. Evenness is quantified using the inverse of Simpson’s index, where a higher value indicates a more even community

In uninoculated soils, remediation did not change community evenness (Fig. 3; acidic soil; coef = 0.32, t-statistic = 1.22, *P* = 0.23, calcareous soil; coef = -0.33, t-statistic = -1.24, *P* = 0.22). However, the addition of inoculant increased the evenness of AM fungal communities in remediated soils relative to unremediated soils (acidic soil; coef = 0.84, t-statistic = 3.48, *P* < 0.001, calcareous soil; coef = 1.29, t-statistic = 5.16, *P* < 0.001), contrasting with the dynamics seen for OTU richness where inoculation dampened the effect of remediation.

### Annual fluctuations in dominant taxa identity

Whilst there was no overall difference in evenness between AM fungal communities in remediated and unremediated soils, inspection of the species rank-abundance curves (across the duration of the study) showed that unremediated communities were generally more heavily dominated by a single OTU compared to those that had undergone remediation, most visible in the calcareous soils. Furthermore, addition of inoculant increased the relative abundance of OTUs occupying intermediate ranks in the community, likely resulting in the overall more even communities as shown above (Fig. 3).

**Fig. 4:**
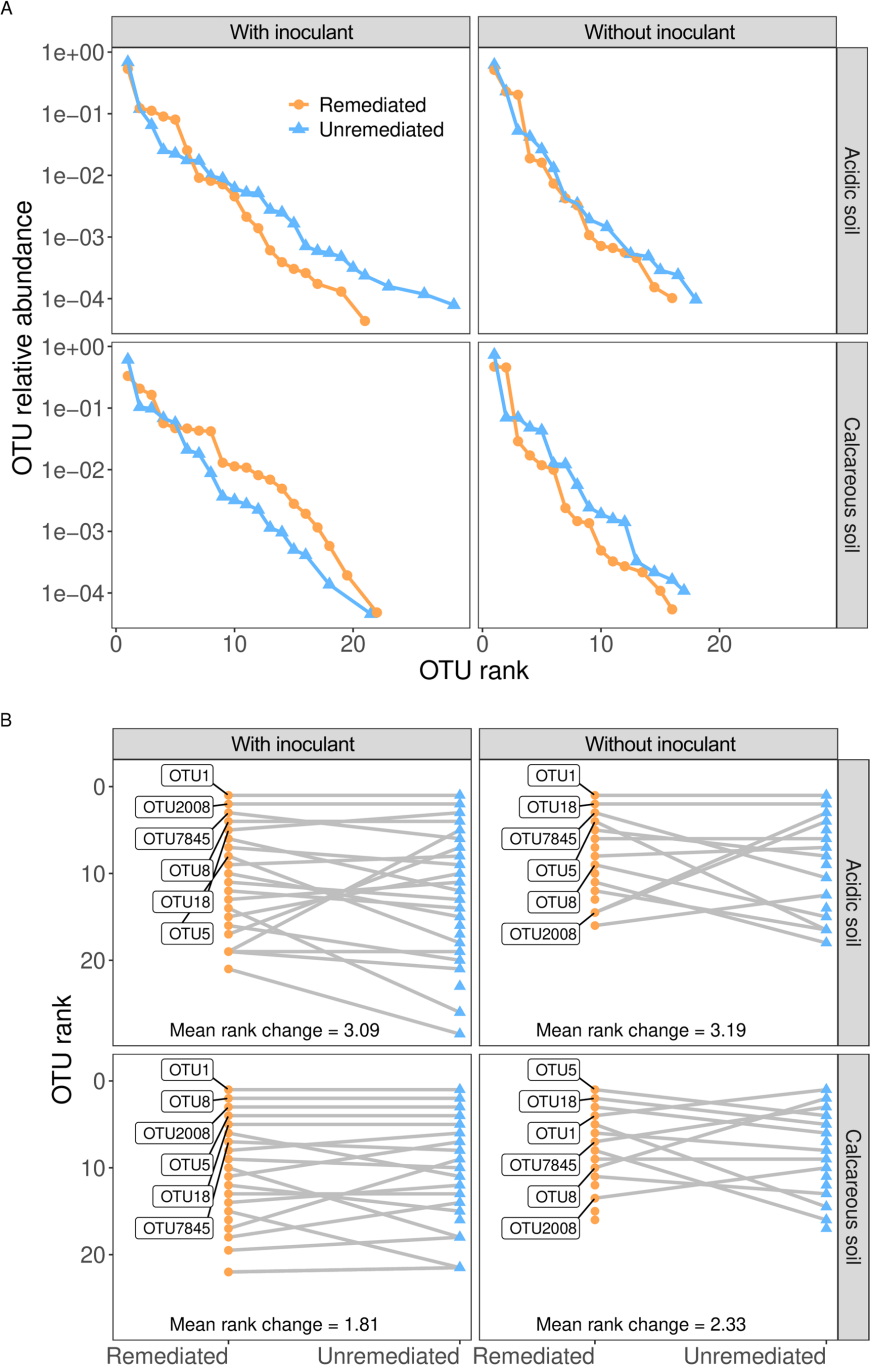
(**A**) Rank-abundance curves for AM fungal communities in each treatment. To calculate these curves, AM fungal communities from individual timepoints within each treatment group were summed together. (**B**) Changes in OTU ranks between AM fungal communities from remediated and unremediated soil-types and inoculation treatments. Mean rank changes show the average change in OTU ranks between remediated and unremediated communities

To further investigate the differences in community structure resulting from remediation and inoculation treatments, we examined the identity and abundances of OTUs that occupy the lowest ranks (e.g. the most abundant OTUs). Notably, the addition of inoculant resulted in a lower mean rank change between remediated and unremediated communities (acidic soil = 3.09, calcareous soil = 1.81), especially in the calcareous soils where, overall, the most abundant four OTUs maintained their ranks regardless of remediation (Fig. 4). In comparison, uninoculated communities showed a higher mean rank change (acidic soil = 3.19, calcareous soil = 2.33), with calcareous soils in particular showing a great degree of rank shuffling even among the most abundant OTUs (Fig. 4).

**Fig. 5:**
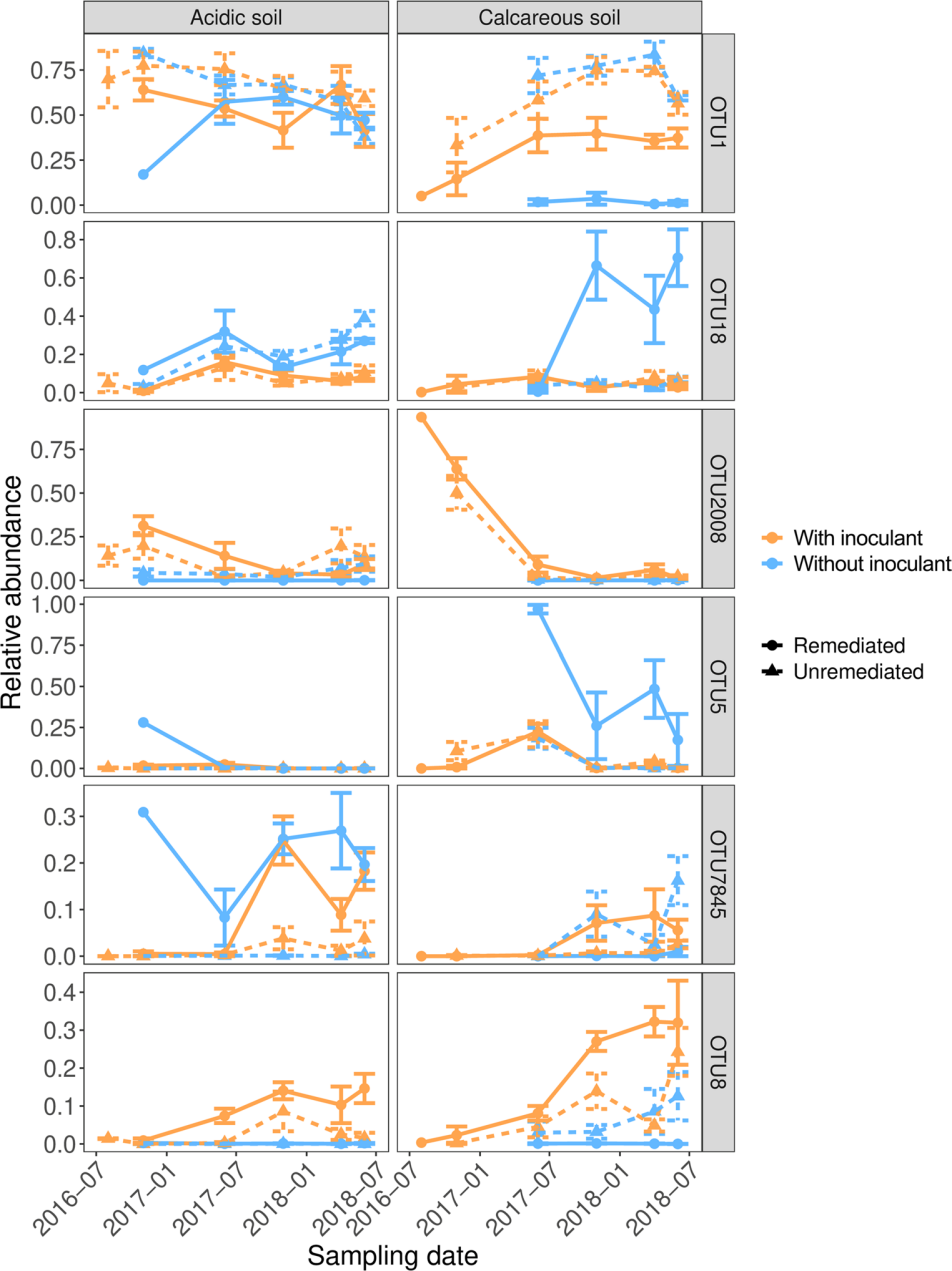
Temporal dynamics of the mean relative abundance of specific AM fungal operational taxonomic units (OTUs) across remediation and inoculation treatments

A more detailed view of individual OTU abundances revealed that specific dominant OTUs were impacted by both remediation and inoculation treatments (Fig. 5). In particular, OTU 1 (100% identical to VT114 - Glomus MO-G17 AM849267, clusters with *Rhizophagus irregularis*) which was the most abundant OTU across our study, was significantly more abundant in unremediated versus remediated soils in both acidic (coef = 1.69, z-statistic = 36.93, *P* < 0.001) and calcareous soils (coef = 24.70, z-statistic = -94.99, *P* < 0.001). In the acidic soils, the addition of inoculant had no significant effect on the relative abundance of OTU 1 in remediated soil (coef = 1.01, z-statistic = 0.56, *P* = 0.57) but increased its relative abundance in unremediated soil (coef = 1.25, z statistic = 11.11, *P* < 0.001). In contrast, adding inoculant to unremediated calcareous soil slightly decreased the relative abundance of OTU 1 (coef = 0.70, z-statistic = -15.68, *P* < 0.001), but dramatically increased its relative abundance in unremediated soils (coef = 42.03, z-statistic = 58.65, *P* < 0.001), highlighting that inoculation with ‘native’ soil can restore the abundance of naturally dominant OTUs after remediation treatment.

The reduced dominance of OTU 1 in remediated calcareous soils that were not subject to inoculant addition meant that other OTUs became relatively more abundant despite being rare in other treatments. For example, OTU 18 (98.6% similar to VT143 - *Glomus* MO-G20 AM849290) was significantly more abundant in remediated uninoculated soil than in all other treatment combinations (coef > 15.26, z-statistic > 75.96|, *P* < 0.001 for all pairwise comparisons). The same trend was also observed for OTU 5 (99.4% similar to VT67 - *Glomus mosseae* AJ306438) which, along with OTU 18, dominated in remediated soils without inoculation (coef > 64.97, z-statistic > 75.98|, *P* < 0.001 for all pairwise comparisons).

Of the OTUs detected in the inoculant (Table S5), few appeared to be significantly more abundant in the soils treated with inoculant. OTU 8 (99.8% similar to VT165 - *Glomus* sp. ​​EF154349), which was a relatively minor component of the inoculant community (mean relative abundance in inoculum = 0.3 ± 0.2%), showed a higher relative abundance in inoculated soils regardless of their remediation treatment in both acidic (remediated; coef = 132, z-statistic = 20.63, *P* < 0.001, unremediated; coef = 138, z-statistic = 9.82, *P* < 0.001) and calcareous soil (remediated; coef = 1096.6, z-statistic = 17.13, *P* < 0.001, unremediated; coef = 1.79, z-statistic = 15.72, *P* < 0.001). OTU 2008 (98.6% similar to VT108 - *Glomus Whitfield type 7* AY330278) was a larger component of the inoculant community compared to OTU 8 (mean relative abundance in inoculant = 23 ± 15%) and showed a similar higher abundance in inoculated soil across our treatment combinations (acidic soil - remediated; coef = 588.2, z-statistic = 12.74, *P* < 0.001, unremediated; coef = 2.41, z-statistic = 22.71, *P* < 0.001, calcareous soil - remediated; coef = 167.1, z-statistic = 11.42, *P* < 0.001, unremediated; coef = 24.7, z-statistic = 13.43, *P* < 0.001), albeit with a different temporal trend with its abundance declining over time.

## Discussion

This study represents the first attempt to revitalise and follow the development of AM fungal communities in two types of remediated soils by reintroducing a vital functional component of the soil biota, the AM fungi, as native (indigenous) inoculants and assessing in detail the diversity of the reintroduced AM fungi. The establishment of diverse AM fungal communities in symbiotic association with host plants is of urgent importance for the restoration of both biological activity and structural integrity of the remediated soil, as only diverse soil biota can restore all ecosystem functions of the original substrate. In addition, using a native (indigenous) mycorrhizal fungi instead of a commercial mycorrhizal inoculant has several advantages, including cost efficiency and avoiding all the negative aspects of commercial inoculants that have been addressed in recent reports (e.g. Hart et al. 2018; Vahter et al. 2023; Koziol et al. 2024). Therefore, in this study we tested whether the use of an indigenous inoculant can be an efficient nature-based solution for the revitalisation of remediated soils.

In our experiment, signs of the first AM fungal colonisation of *Lolium perenne* roots were detected in two experimental mesocosms already during the first growing season, only one month after the start of the experiment (Maček et al. 2022). One year and five months after the start of the experiment, mycorrhizal colonisation in the root system was visible and present in *Lolium* roots in all treatments. The intensity of mycorrhizal colonisation in the root system was significantly different in both non-remediated soils (calcareous and acidic) compared to the remediated soils, showing a clear effect of the remediation process on AM fungal root colonisation. Illumina sequencing revealed 36 OTUs in the experimental treatments matching 33 unique MaarjAM virtual taxa (VT) (Öpik et al. 2010), with the majority (81%) belonging to the Glomeraceae family and 6% to each of the Acaulosporaceae and Claroideoglomeraceae families, with single OTUs from each of the Diversisporaceae, Paraglomeraceae, and Archaeosporaceae families. A similar diversity (taxa richness) is found in semi-natural temperate grassland systems (e.g. Maček et al. 2019), while 17 OTUs were identified in the rhizosphere of the grassland used as a native inoculant in this study (Table S5), including 14 Glomeraceae OTUs, one Acaulosporaceae, one Archeosporaceae and one Claroideoglomeraceae OTU. This indicates that 22 OTUs that were not identified in the native inoculant used were either present in the unremediated soil or were introduced into the treatments by different potential dispersal methods (e.g., wind, water, and/or animal vectors).

### AM fungal community composition

The first hypothesis of our study tested whether EDTA washing (remediation), and inoculation affect the composition of AM fungal communities in the different soil types in different ways, resulting in different AM fungal communities, and whether the effects on community composition after remediation were smaller when the inoculant was added. In the two polluted soil types, AM fungal community composition differed significantly between the remediation and inoculant treatments (Fig. 1). In acidic soils, remediation had a significant effect on community composition (*P* < 0.001), accounting for 31.5% of the variation in composition, while in calcareous soils, remediation had a smaller but also significant (*P* < 0.01) effect on AM fungal community composition. Previous studies show that the composition of AM fungal communities in environmental samples is largely determined by edaphic factors, particularly soil pH and soil type (Dumbrell et al. 2010a; Hazard et al. 2013). Temperature and pH are considered the most important abiotic factors in soil that explain the global distribution of AM fungi, while the ecological niches of AM fungi are still poorly understood (Davison et al. 2021). In our experiment, soil pH could influence the development of AM fungal community composition in remediated and unremediated soils, with greater differences between initial (before, pH = 5.0) and final (after remediation, pH = 6.2; Table S1) soil pH in acidic soil. In addition, the unremediated soils had a different original use at the time of soil sampling, i.e. an active vegetable garden in the case of the Slovenian calcareous soil and an abandoned field (secondary grassland) in the case of the Austrian acidic soil, which could influence the AM fungal communities in unremediated treatments. Nevertheless, there is still little detailed information on the organism–environment relationships between the AM fungal taxa at approximate species level (Davison et al. 2021), and this needs further investigation. The addition of an indigenous inoculant reduced the differences in composition between remediated and unremediated soils, both in calcareous and acidic soils (to a lesser extent in the latter), thereby reducing the effect of remediation (Fig. 1). Only in calcareous soils did the addition of inoculant also reduce the compositional dispersion of communities in the remediated soils. The reason that the effect of the inoculant was more pronounced in calcareous soils could be that the inoculant came from a non-contaminated calcareous grassland near the experimental site, so that the soil properties of inoculant, especially the pH, were closer to the calcareous soils used for remediation. The compatibility of the introduced species with the local conditions, was counted among the factors determining the success of the inoculation, alongside the priority effects and the carrying capacity of the soil (habitat quality), the frequency of the AM fungi and the compatibility of the species (Verbruggen et al. 2013).

### AM fungal diversity

To test our second hypothesis, the effects of remediation and inoculation on the diversity of AM fungi in the two different soil types and the temporal changes in diversity with increasing time after remediation were analysed. In acidic soils without inoculation, remediation reduced OTU richness (*P* < 0.001) (Fig. 2). The addition of the inoculant cancelled out the differences in richness between remediated and unremediated soils and increased richness in both soil treatments by ~ 43% (*P* < 0.001). However, the situation was different in the calcareous soils, where remediation had no effect on OTU richness in either inoculation treatment, although inoculation still reduced the difference in OTU richness between remediated and unremediated soils (Fig. 2). In contrast to OTU richness, remediation did not alter overall community evenness (Fig. 3). However, the addition of an inoculant increased the evenness of AM fungal communities in remediated soils compared to unremediated soils, which contrasts with the dynamics observed for OTU richness, where inoculation reduced the effect of remediation. The effects of physical disturbance on AM fungal communities have been measured previously (Lekberg et al. 2012) and while disturbances in the different soil treatments on arable land had no effect on the composition of AM fungal communities, priority effects were proposed to be responsible for the differences in communities following disturbance. In the uninoculated remediated soils, the initial number of AM fungal OTUs is limited to those that can efficiently disperse from the environment, probably mainly by wind dispersal, as it is very unlikely that any AM fungi could survive the EDTA washing treatment. Therefore, the development of community composition in remediated and non-inoculated treatments may depend entirely on priority effects (order of arrival), i.e. the fungal taxa that arrive first and can efficiently associate with plant roots will dominate the community. Previous studies have shown that during the plant-growth period AM fungal communities consistently display a close fit to a log-normal species abundance distribution, most likely due to a niche-based community assembly mechanism, but with a clear overdominance of the most abundant taxon, which on average accounts for 40% of the total abundance within the community (Dumbrell et al. 2010b) – a pattern also observed in our study (Fig. 4). This may change in the next season, assuming the seasonal changes in community evenness that may be caused by changes in the competitive dynamics of AM fungi as carbon supply increases during the warmer growing season, as suggested by Dumbrell et al. (2010b). However, the identity of the taxon that acquires dominance in natural ecosystems may be largely stochastic (Dumbrell et al. 2010b) and, in the case of non-inoculated remediated soils, may depend largely on priority effects and dispersal from surrounding ecosystems. Stochasticity in the order of arrival has been cited as an important determining factor in several studies on AM fungal communities (e.g. Dumbrell et al. 2011; Caruso et al. 2012). This principle, which affects fungal community composition, could be particularly important in remediated soils, which, according to the harsh EDTA clean-up protocols, are more or less an empty space with no or a very limited original community. Thus, if the introduced AM fungi can reach unoccupied areas earlier than other competing species via inoculants or by different dispersal methods (e.g. by wind or animal dispersal), they may benefit from priority effects (see the rank changes in AM taxa depending on remediation and inoculant addition in Fig. 4). This means that priority effects can significantly influence the composition of AM fungal communities in remediated soils, especially in the first year of community establishment (Fig. 5), as the order of arrival influences the success of different AM fungal species in colonising a host plant. The strength of priority effects may depend on both host plant identity and neighbouring plant species, suggesting that plant interactions play a crucial role in AM fungal community formation (Hausmann and Hawkes, 2010; Werner and Kiers 2015). In our experiment a monoculture of the grass species *Lolium perenne* was selected as a single host plant in order to ensure a homogeneous rhizosphere in all treatments and to minimise the effect of plant species.

### AM fungal taxa identity

We partially confirmed the third hypothesis that disturbance (remediation) and inoculation lead to a shift in the identity of dominant taxa reflecting different traits among the AM fungi in the soils studied. We identified 36 OTUs in the experimental treatments that matched 33 unique MaarjAM virtual taxa (VT), with the majority of taxa belonging to the Glomeraceae family (81%). Unremediated communities were generally more dominated by a single OTU than those that had undergone remediation as shown by the rank-abundance curves (over the entire duration of the study). The addition of inoculant increased the relative abundance of OTUs occupying intermediate ranks in the community, likely resulting in the more evenly distributed communities shown in Fig. 3. A more detailed analysis of individual OTU abundances revealed that certain dominant OTUs were affected by both the remediation and inoculation treatments (Figs. 4 and 5). In particular, OTU 1 (clusters with *Rhizophagus irregularis*, 100% identical to VT 114 - *Glomus* (MO-G17) AM849267) was significantly more abundant in unremediated soils than in remediated soils. In calcareous soils, the addition of the inoculant did not significantly change the abundance of OTU 1 in unremediated soils but dramatically increased its relative abundance in remediated soils (Fig. 4). This is as expected as OTU 1 was present with the highest relative abundance of 0.42 ± 0.21 (Table S5) in the inoculant and therefore could develop in the remediated soil at the beginning of the experiment with limited competition in the remediated substrate. Thus, inoculation with ‘native’ soil can restore the abundance of naturally abundant OTUs after the remediation treatment. This is particularly important as OTU 1 (*R. irregularis*) has previously been reported as an AM taxon that is sensitive to disturbance and normally occurs in soils that are less affected by human activities (e.g. Helgason et al. 1998). In a study investigating whether the function of mycorrhiza is influenced by the quantitative composition of the fungal community, Blažková et al. (2021) reported that most AM fungal communities formed in a pot experiment from artificial inoculant pools (synthetic communities) differed both in composition and function from the communities with spontaneously formed ratios of AM fungal species. An exception was the synthetic community enriched with *R. irregularis*, which had almost the same composition as the spontaneously established AM fungal community and induced very similar responses in the host plant. Root colonisation tended to be higher in the *R. irregularis*-enriched community than in the other communities (Blažková et al. 2021), ​strongly suggesting that other isolates developed less intraradical biomass than *R. irregularis*. This also explains its dominance and high species rank in the community when assessed from root (and not soil) samples, which was also the case in our study.

In our experiment, calcareous soils without inoculant, OTU 5 and OTU 18 dominated the community, especially towards the end of the study (Figs. 4 and 5). Interestingly, in the cases where OTU 1 did not dominate (e.g. in remediated calcareous soils to which no inoculant was added), other OTUs were relatively abundant, although they were less common in other treatments. In particular, OTU 18 (98.6% similar to VT143 - *Glomus* MO-G20 AM849290) was significantly more abundant in remediated, uninoculated soil than in all other treatment combinations. The same trend was observed for OTU 5 (99.4% similar to VT67 - *Funneliformis mosseae* AJ306438), which together with OTU 18 dominated in uninoculated remediated soils. *F. mosseae* is considered an AM fungus with a fast life cycle (Chagnon et al. 2013; Oehl et al. 2004, 2009). Blažková et al. (2021) report that the plateau of its infectivity in the soil is lower than that of *R. irregularis* or that it develops less root colonisation than *Rhizophagus* at the same density of infectious propagules. The latter assumption is supported by several earlier studies (Janoušková et al. 2013; Thonar 2009; Wagg et al. 2011), in which *F. mosseae* and *Claroideoglomus claroideum* developed less root colonisation than *R. irregularis* from standardised numbers of infectious propagules.

Therefore, there are probably two main mechanisms behind the community development in remediated soils in the first year of the experiment: (1) priority effects or order of arrival and (2) competitive exclusion among the dominant taxa (i.e. OTU 5, OTU 18 and OTU 1). *F. mosseae* is known to be an efficient phosphate supplier, but it does not become or remain more abundant in communities due to higher carbon supply from the host plant in a co-colonising AM fungal community. The answer is probably related to the rapid growth and/or high soil infectivity of *R. irregularis*, as differences in growth rates between co-colonising AM fungal species are likely to reduce the efficiency of preferential carbon allocation (Werner and Kiers 2015). The traits of *R. irregularis* may have enabled it to capture a large amount of carbon from the plant’s initial investment into the symbiosis (Bever 2015; Christian and Bever 2018) and to become dominant within the community by the mechanism of competitive exclusion and as the *R. irregularis* was the dominant taxon in our native inoculant it was also dominating in inoculated treatments (see OTU rank shifts in Fig. 4). *F. mosseae* belongs to a group of AM fungi characterised by a ruderal or opportunistic ecological strategy, as evidenced by findings that *F. mosseae* is frequently located in the form of spores in natural soils and trap cultures, yet is less commonly found in the roots of plants from natural environments (Öpik et al. 2014). Moreover, it has been reported that *F. mosseae* tends to exhibit increased abundance in various disturbed habitats. Helgason et al. (2007) proposed that competitive release might account for the observed abundance of *F. mosseae* subsequent to the application of the fungicide benomyl, which altered the community dynamics of AM fungi within undisturbed soil monoliths. Maček et al. (2016) studying EDTA washed contaminated soil in a pot experiment found that *F. mosseae*-related OTUs, specifically those aligning with MaarjAM virtual taxa VT67 and VT265, were only detected in treatments characterised by a relatively low diversity of AM fungal taxa. The relative abundance of *F. mosseae* decreased inversely with increasing richness of AM fungal taxa in plant roots and notably, also in the study of Maček et al. (2016) F. *mosseae* was absent in environmental grassland root samples, including roots of common mycorrhizal plant *Plantago lanceolata*.

## Conclusion

The remediation process has a significant effect on AM fungal communities in the remediated soil. The use of native inoculants in remediated soils leads to the establishment of diverse AM fungal communities, including those taxa that are abundant and dominant in roots in natural environments, such as *Rhizophagus irregularis* (OTU 1), as well as some rare taxa that provide an opportunity and a boost for spontaneous establishment of an AM fungal community in disturbed soils that are closer in composition to communities in untreated soil. Mycorrhizal symbioses established from natural AM fungal compositions have been shown to be more beneficial to the host-plant than those from artificially manipulated synthetic communities (Blažková et al. 2021). Therefore, in the case of revitalisation of remediated soil we once again argue that the use of native (indigenous) inoculants from similar ecosystems (in this case extensively managed grassland soil of a similar pH) is the best way to establish diverse and functionally efficient communities in soils following heavy metal remediation. This emphasises the importance of nature-based solutions in establishing functional communities of plant-symbiotic AM fungi in soils that have been exposed to different disruptions affecting their diversity, including conventional agriculture systems and urban soils.

## Supplementary Information

Below is the link to the electronic supplementary material.

Supplementary Material 1

## Data Availability

All data and R code required to reproduce our statistical analyses have been deposited into a Figshare collection https://figshare.com/s/7cc6da4340724cb3c8c1. Raw sequences are deposited in GenBank under BioProject ID PRJNA1347864.
