# Supplementary material for: Indigenous inoculant dampens the impact of remediation of heavy metal polluted soil on arbuscular mycorrhizal fungal communities
Source: Mycorrhiza. 2026 May 9;36(3):23. doi: 10.1007/s00572-026-01268-1 (PMC13156140; doi:10.1007/s00572-026-01268-1)
Supplement: Supplementary file 1 — Supplementary Material 1 [file 572_2026_1268_MOESM1_ESM.pdf]

SUPPLEMENTAL MATERIAL

Indigenous inoculant dampens the impact of remediation of heavy metal polluted soil on arbuscular mycorrhizal fungal communities

Table S1. Soil parameters at the start of the experiment.

Table S2. Metal content in plant tissue sampled on 9 November 2016.

Table S3. Plant biomass (g dry weight) measured at 1, 4, and 11 months after planting.

Figure S4. Rarefaction curves.

Table S5. Details of AM fungal taxa detected in inoculant soils arranged in order of decreasing relative abundance.

Table S1. Soil parameters at the start of the experiment (summer 2016) measured in untreated (unremediated) and remediated soils from two sites (acidic soil from Austria and calcareous soil from Slovenia). Values represent means ± standard error (SE) for heavy metal concentrations (Cd, Pb, Zn); different letters indicate significant differences among treatments based on one-way ANOVA followed by Tukey's HSD test ($P < 0.05$).

	Acidic unremediated	Acidic remediated	Calcareous unremediated	Calcareous remediated
Cd [mg/kg]	4.28 ± 0.07^{a}	0.83 ± 0.06^{b}	6.74 ± 0.08^{c}	3.07 ± 0.15^{d}
Pb [mg/kg]	756.04 ± 12.09^{a}	202.72 ± 1.25^{b}	1131.20 ± 27.12^{c}	479.54 ± 13.86^{d}
Zn [mg/kg]	452.95 ± 7.06^{a}	262.08 ± 2.22^{b}	755.97 ± 14.75^{c}	513.64 ± 20.84^{d}
texture	loam	Silt loam	Sandy loam	Silt loam
P_2O_5[mg/100g]	2.6	8.5	185.5	192.4
K_2O[mg/100g]	10.9	23.1	42.2	26.3
Org. matter [%]	5.2	4.9	11.4	7.0
C [%]	3.0	2.8	6.6	4.1
N [%]	0.24	0.23	0.49	0.40
C / N	12.5	12.2	13.5	10.2
pH	5.0	6.2	6.9	7.3

Table S2. Metal content in plant tissue sampled on 9 November 2016. Data are presented as mean ± standard error (SE). Different letters indicate significant differences among treatments based on one-way ANOVA followed by Tukey’s HSD test ($p < 0.05$). Treatments include untreated (unremediated) and remediated soils from two sites (acidic soil from Austria and calcareous soil from Slovenia), with and without arbuscular mycorrhizal fungal inoculation. Elements are reported in ppm, ppb, or %, as indicated.

		Acidic	Acidic	Acidic	Acidic	Calcareous	Calcareous	Calcareous	Calcareous
		unremediated	unremediated	remediated	remediated	unremediated	unremediated	remediated	remediated
			+inoculant		+inoculant		+inoculant		+inoculant
Mo	PPM	3.19 ± 0.36b	2.3 ± 0.23^{b}	4.76 ± 0.35^{b}	4.68 ± 0.27^{b}	16.55 ± 1.09^{a}	19.34 ± 1.03^{a}	4.36 ± 0.23^{b}	4 ± 0.65^{b}
Cu	PPM	10.84 ± 0.53	9.9 ± 0.31	9.67 ± 0.51	8.19 ± 0.6	8.04 ± 0.69	7.88 ± 0.38	9.19 ± 1.44	9.55 ± 0.55
Pb	PPM	22.63 ± 2.56^{a}	22.9 ± 0.79^{a}	2.14 ± 0.5^{b}	2.11 ± 1.2^{b}	5.98 ± 2.18^{b}	4.28 ± 1.12^{b}	4.51 ± 0.33^{b}	3.2 ± 1.16^{b}
Zn	PPM	340.22 ± 11.64^{a}	354.95 ± 23.09^{a}	54.83 ± 1.87^{b}	41.27 ± 2.75^{b}	73.22 ± 5.02^{b}	68.85 ± 6.76^{b}	48.77 ± 2.97^{b}	49.05 ± 5^{b}
Ag	PPB	11.5 ± 0.87^{a}	13 ± 0.91^{a}	3.25 ± 0.25^{b}	2.75 ± 1.03^{b}	3.75 ± 0.48^{b}	3 ± 0^{b}	3.75 ± 0.25^{b}	2.75 ± 0.48^{b}
Ni	PPM	3.83 ± 0.37	4.38 ± 0.41	2.78 ± 0.77	3.2 ± 1.94	2.58 ± 0.17	2.62 ± 0.37	2.25 ± 0.17	1.8 ± 0.41
Co	PPM	0.17 ± 0.03	0.17 ± 0.03	0.22 ± 0.03	0.21 ± 0.09	0.12 ± 0.02	0.13 ± 0.02	0.15 ± 0	0.12 ± 0.03
Mn	PPM	80.5 ± 7.1^{b}	105.75 ± 5.17^{a}	46 ± 6.03^{c}	35.75 ± 3.71^{cd}	15.75 ± 1.65^{e}	15 ± 1.41^{e}	22.25 ± 1.18^{de}	19.75 ± 3.2^{de}

Fe	%	38.25 ± 7.6	38.5 ± 8.43	45 ± 7.95	42 ± 20.6	27 ± 4.6	24 ± 3.24	34.25 ± 2.39	28.25 ± 7.31
As	PPM	0.25 ± 0.06	0.28 ± 0.11	0.28 ± 0.13	0.3 ± 0.14	0.03 ± 0.03	0 ± 0	0.03 ± 0.03	0 ± 0
Au	PPB	0.65 ± 0.15^{a}	0.45 ± 0.26^{ab}	0.12 ± 0.08^{ab}	0.15 ± 0.1^{ab}	0.12 ± 0.08^{ab}	0.05 ± 0.05^{b}	0.1 ± 0.06^{ab}	0.15 ± 0.1^{ab}
Th	PPM	0.04 ± 0.01	0.04 ± 0.01	0.04 ± 0.01	0.04 ± 0.03	0.01 ± 0	0.01 ± 0.01	0.02 ± 0	0.02 ± 0.01
Sr	PPM	19.98 ± 0.8^{a}	19.73 ± 0.71^{a}	7.2 ± 0.53^{b}	6.97 ± 0.66^{b}	9.53 ± 0.26^{b}	10.15 ± 0.87^{b}	9.88 ± 0.24^{b}	9.53 ± 1.08^{b}
Cd	PPM	2.88 ± 0.12^{a}	3.08 ± 0.22^{a}	0.19 ± 0.03^{b}	0.12 ± 0.01^{b}	0.3 ± 0.04^{b}	0.29 ± 0.04^{b}	0.14 ± 0.01^{b}	0.15 ± 0.02^{b}
Sb	PPM	0.18 ± 0.02	0.16 ± 0.03	0.18 ± 0.02	0.17 ± 0.06	0.28 ± 0.1	0.13 ± 0.02	0.16 ± 0.01	0.12 ± 0.02
V	PPM	3.5 ± 0.29	3 ± 0	4.5 ± 1.66	5.25 ± 2.63	4.5 ± 0.29	3.75 ± 0.48	4.25 ± 0.25	3.25 ± 0.75
Ca	%	0.7 ± 0.02^{ab}	0.68 ± 0.02^{ab}	0.69 ± 0.06^{ab}	0.62 ± 0.03^{b}	0.63 ± 0.01^{b}	0.64 ± 0.02^{ab}	0.78 ± 0.03^{a}	0.76 ± 0.04^{ab}
P	%	242.75 ± 12.4^{bc}	249.25 ± 8.22^{bc}	198.75 ± 15.88^{c}	227.25 ± 3.47^{c}	346.5 ± 15.42^{a}	325.75 ± 12.66^{a}	298.75 ± 15.4^{ab}	313 ± 8.73^{a}
La	PPM	0.23 ± 0.05	0.24 ± 0.04	0.15 ± 0.03	0.16 ± 0.1	0.06 ± 0.01	0.07 ± 0.03	0.09 ± 0.01	0.07 ± 0.02
Cr	PPM	6.55 ± 0.82	6.15 ± 0.21	9.4 ± 2.48	10 ± 4.81	8.32 ± 0.41	7.47 ± 1.19	7.55 ± 0.42	5.88 ± 1.16
Mg	%	244.75 ± 6.16^{a}	232.75 ± 7.22^{abc}	176.75 ± 5.22^{d}	168 ± 7.2^{d}	258.25 ± 13.01^{a}	235.5 ± 10.53^{ab}	202.75 ± 7.34^{bcd}	197.25 ± 4.07^{cd}
Ba	PPM	190.6 ± 6.07^{a}	189.7 ± 10.94^{a}	52.05 ± 4.66^{b}	54.15 ± 9.68^{b}	27.5 ± 2.16^{bc}	32.7 ± 4.87^{bc}	21.68 ± 0.83^{c}	22.35 ± 3.71^{c}

Ti	PPM	5.5 ± 0.65	6.75 ± 1.8	6 ± 0.91	6 ± 2.04	6.5 ± 0.96	8 ± 2.42	8 ± 0.41	6.75 ± 1.55
B	PPM	4.25 ± 0.25	4 ± 0	4.25 ± 0.48	4 ± 0	4.5 ± 0.29	4.5 ± 0.29	4.5 ± 0.29	4.5 ± 0.29
Al	%	0.02 ± 0	0.02 ± 0.01	0.02 ± 0	0.02 ± 0.01	0.01 ± 0	0 ± 0	0.01 ± 0	0.01 ± 0
Na	%	27.75 ± 3.42[b]	30.75 ± 1.75[b]	156.75 ± 25.24[a]	149.5 ± 23.89[a]	23 ± 1.87[b]	22.75 ± 1.7[b]	122.25 ± 27.83[ab]	189 ± 53.13[a]
K	%	1.75 ± 0.06	1.81 ± 0.03	2.08 ± 0.4	1.91 ± 0.15	2.07 ± 0.09	1.96 ± 0.25	1.91 ± 0.15	1.97 ± 0.26
Sc	PPM	0.22 ± 0.02	0.22 ± 0.02	0.22 ± 0.02	0.22 ± 0.02	0.25 ± 0.03	0.2 ± 0	0.22 ± 0.02	0.2 ± 0.04
Tl	PPM	0.43 ± 0.02[b]	0.54 ± 0.04[a]	0.17 ± 0.03[c]	0.12 ± 0.01[c]	0 ± 0[d]	0 ± 0[d]	0.03 ± 0[d]	0.01 ± 0.01[d]
S	%	0.34 ± 0.01[a]	0.32 ± 0.01[a]	0.32 ± 0.03[a]	0.31 ± 0.02[ab]	0.26 ± 0.01[ab]	0.26 ± 0.02[ab]	0.23 ± 0.01[b]	0.26 ± 0.01[ab]
Hg	PPB	18.25 ± 1.03	18.5 ± 1.71	20 ± 1.22	17.25 ± 1.31	16.5 ± 0.87	16.25 ± 1.25	18.25 ± 0.85	19.5 ± 1.71
Se	PPM	0.4 ± 0	0.4 ± 0	0.45 ± 0.03	0.43 ± 0.02	0.4 ± 0	0.4 ± 0	0.38 ± 0.03	0.45 ± 0.03

Table S3. Plant biomass (g dry weight) measured at 1, 4, and 11 months after planting. Values represent means ± standard error (SE). Different letters within each time point indicate statistically significant differences among treatments based on one-way ANOVA followed by Tukey's HSD test (p < 0.05). Treatments include untreated (unremediated) and remediated soils from Austria (acidic soil) and Slovenia (calcareous soil), with and without arbuscular mycorrhizal fungal inoculation.

	After 1 month	After 4 month	After 11 month
Acidic unremediated	0.51 ± 0.28^{d}	9.45 ± 0.22^{d}	11.93 ± 2.05^{bc}
Acidic unremediated + inoculant	0.59 ± 0.15^{cd}	11.25 ± 0.76^{d}	8.09 ± 1.16^{c}
Acidic remediated	1.63 ± 0.15^{a}	25.1 ± 1.45^{a}	19.91 ± 1.57^{b}
Acidic remediated + inoculant	1.31 ± 0.19^{ab}	21.29 ± 1.71^{ab}	21.09 ± 2.60^{b}
Calcareous unremediated	1.0 ± 0.14^{bcd}	13.81 ± 1.14^{cd}	20.4 ± 3.00^{b}
Calcareous unremediated + inoculant	1.16 ± 0.12^{ab}	10.12 ± 1.05^{d}	13.995 ± 0.16^{bc}
Calcareous remediated	1.48 ± 0.23^{ab}	17.21 ± 3.72^{bc}	44.71 ± 9.88^{a}
Calcareous remediated + inoculant	1.03 ± 0.07^{bc}	21.17 ± 1.22^{ab}	9.67 ± 0.89^{bc}

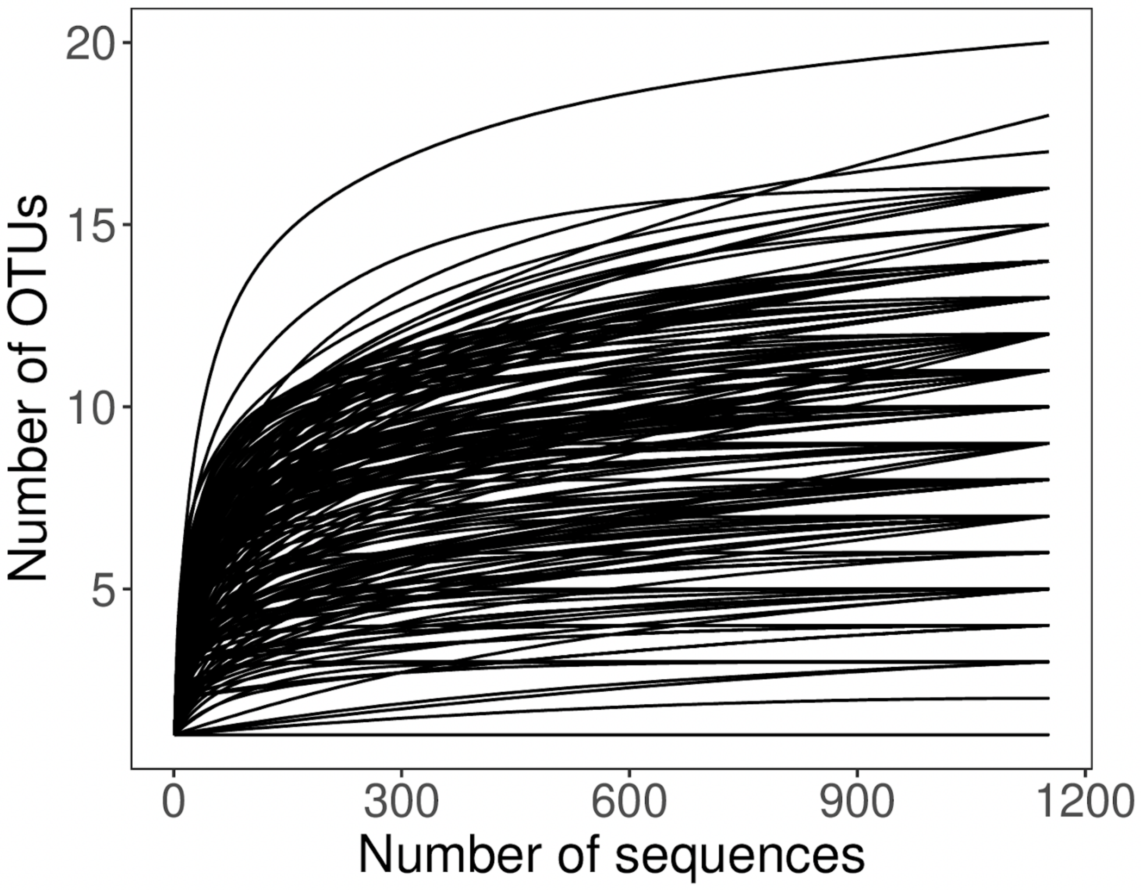

Figure S4. Rarefaction curves showing the relationship between sequencing depth (number of sequences) and observed arbuscular mycorrhizal fungal diversity (number of OTUs) across samples. Each line represents a single sample.

Table S5. Details of AM fungal taxa detected in inoculant soils arranged in order of decreasing relative abundance. Each virtual taxon (VT) was represented by a single OTU in the inoculum samples.

MaarjAM virtual taxon (VT)	Taxonomy and accession number of type VT	OTU (percentage similarity to VT type-sequence in MaarjAM database	Mean and std error relative abundance in inoculant
00114	Glomus (MO-G17) AM849267	OTU 1 (100%)	0.42 ± 0.21
00108	Glomus (Whitfield type 7) AY330278	OTU 2008 (98.6%)	0.23 ± 0.15
00309	Glomus (Orvin GLO3E) FJ194511	OTU 30 (97.7%)	0.10 ± 0.05
00143	Glomus (MO-G20) AM849290	OTU 18 (98.6%)	0.06 ± 0.03
00135	Glomus (MO-G15) AM849273	OTU 28 (99.6%)	0.05 ± 0.04
00310	Glomus (Orvin GLO3D) FJ194510	OTU 13 (97.9%)	0.05 ± 0.05
00214	Glomus (GLO7) AF074370	OTU 17 (99.0%)	0.04 ± 0.01
219	Glomus (MO-G5) AM849279	OTU 34 (98.8%)	0.03 ± 0.02
222	Glomus (MO-G23) AM849264	OTU 91 (100%)	0.01 ± 0.01
125	Glomus (MO-G22) AM849263	OTU 5991 (98.1%)	0.005 ± 0.005
276	Claroideoglomus (Glo59) EF041095	OTU 377 (99.0%)	0.003 ± 0.003
165	Glomus sp. EF154349	OTU 8 (99.8%)	0.003 ± 0.002
156	Glomus (Wirsel OTU16) AJ563861	OTU 7845 (98.4%)	0.001 ± 0.001
010	Acaulospora (Acau4) AF074350	OTU 2769 (97.3%)	0.001 ± 0.001
005	Archaeospora (Other1) AF131054	OTU 825 (99.2%)	0.001 ± 0.001
202	Glomus (Wirsel OTU6) AJ563889	OTU 16093 (99.8%)	0.001 ± 0.001
193	Claroideoclomus lamellosum AJ276087	OTU 1784 (100%)	0.001 ± 0.0003
